# Epidermodysplasia Verruciformis: Inborn Errors of Immunity to Human Beta-Papillomaviruses

**DOI:** 10.3389/fmicb.2018.01222

**Published:** 2018-06-12

**Authors:** Sarah J. de Jong, Elias Imahorn, Peter Itin, Jouni Uitto, Gérard Orth, Emmanuelle Jouanguy, Jean-Laurent Casanova, Bettina Burger

**Affiliations:** ^1^St. Giles Laboratory of Human Genetics of Infectious Diseases, Rockefeller Branch, The Rockefeller University, New York NY, United States; ^2^Department of Biomedicine, University Hospital of Basel, University of Basel, Basel, Switzerland; ^3^Department of Dermatology, University Hospital of Basel, Basel, Switzerland; ^4^Department of Dermatology and Cutaneous Biology, Sidney Kimmel Medical College, Thomas Jefferson University, Philadelphia, PA, United States; ^5^Jefferson Institute of Molecular Medicine, Thomas Jefferson University, Philadelphia PA, United States; ^6^Pasteur Institute, Paris, France; ^7^INSERM UMR 1163, Laboratory of Human Genetics of Infectious Diseases, Necker Branch, Necker Hospital for Sick Children, Paris, France; ^8^Imagine Institute, Paris Descartes University, Paris, France; ^9^Pediatric Hematology and Immunology Unit, Necker Hospital for Sick Children, Paris, France; ^10^Howard Hughes Medical Institute, New York NY, United States

**Keywords:** epidermodysplasia verruciformis, beta-HPV, TMC/EVER, non-melanoma skin cancer, primary immunodeficiency

## Abstract

Epidermodysplasia verruciformis (EV) is an autosomal recessive skin disorder with a phenotype conditional on human beta-papillomavirus (beta-HPV) infection. Such infections are common and asymptomatic in the general population, but in individuals with EV, they lead to the development of plane wart-like and red or brownish papules or pityriasis versicolor-like skin lesions, from childhood onwards. Most patients develop non-melanoma skin cancer (NMSC), mostly on areas of UV-exposed skin, from the twenties or thirties onwards. At least half of the cases of typical EV are caused by biallelic loss-of-function mutations of *TMC6/EVER1* or *TMC8/EVER2*. The cellular and molecular basis of disease in TMC/EVER-deficient patients is unknown, but a defect of keratinocyte-intrinsic immunity to beta-HPV is suspected. Indeed, these patients are not susceptible to other infectious diseases and have apparently normal leukocyte development. In contrast, patients with an atypical form of EV due to inborn errors of T-cell immunity invariably develop clinical symptoms of EV in the context of other infectious diseases. The features of the typical and atypical forms of EV thus suggest that the control of beta-HPV infections requires both EVER1/EVER2-dependent keratinocyte-intrinsic immunity and T cell-dependent adaptive immunity.

## Introduction

Epidermodysplasia verruciformis (EV) is a rare skin disease characterized by persistent disseminated flat warts and pityriasis versicolor-like lesions, associated with a high risk of non-melanoma skin cancer (NMSC). About 501 patients have been reported worldwide (Burger and Itin, 2014; Imahorn et al., 2017). EV was first described in Lewandowsky and Lutz (1922) as a congenital skin disease. The observation of parental consanguinity and familial cases led to the suggestion that EV might be a genetic disease with autosomal recessive inheritance (Cockayne, 1933; Rajagopalan et al., 1972). This notion was not confirmed until 70 years later, when inactivating mutations of the *EVER1/TMC6* and *EVER2/TMC8* genes accounting for about 50% of all known cases of EV were identified (Ramoz et al., 2002; Orth, 2006, 2008; Imahorn et al., 2017). Auto- and heteroinoculation experiments performed from 1946 onward showed that the phenotype of EV was dependent on a viral infection (Lutz, 1946; Jablonska and Milewski, 1957). Viral particles were then detected in EV lesions (Ruiter and van Mullem, 1966; Jablonska et al., 1968), and, subsequently, EV-specific human papillomaviruses (HPVs) were identified, some of which, such as HPV-5 in particular, were shown to be associated with the cancers observed in EV patients (reviewed in Orth et al., 1980; Orth, 2006). EV thus results from a genetically determined susceptibility to specific skin-tropic HPVs (Orth, 2006, 2008; Imahorn et al., 2017). These viruses belong to the beta-genus, and are almost ubiquitous and apparently benign in the general population. EV patients are not prone to other viral, bacterial, or fungal infections. NMSC is the only type of cancer presenting a higher incidence in these patients than in the general population (Orth, 2006, 2008). The EVER1 and EVER2 proteins are thought to govern keratinocyte-intrinsic immunity to beta-HPVs. However, patients with cutaneous clinical symptoms of beta-HPV infection and other infectious manifestations caused by profound T-cell defects have been reported. This clinical picture is referred to as “atypical EV." The cumulative findings of almost a century of studies of typical and atypical EV suggest that both keratinocytes and T cells are required for the control of beta-HPVs. We review here the various facets of EV.

## Clinical Manifestations and Histopathology of Ev

EV manifests as multiple polymorphic skin lesions, which begin to appear during infancy or early childhood and persist throughout the individual’s life. Flat-topped papular lesions resembling verrucae planae typically develop on the extremities. The lesions may also present as red or red-brownish papules or pityriasis versicolor-like lesions, mostly on the trunk, neck, and face (Lewandowsky and Lutz, 1922). In addition, verruca-like papillomatous or seborrheic keratosis-like lesions may develop. The frequency of verruca vulgaris is no higher in these patients than in the general population. In many patients, isomorphic lesions develop preferentially at sites of traumas (the Koebner phenomenon) (de Oliveira et al., 2003). Some patients have persistent palmar lesions similar to palmar pits and dermatoscopically consistent with plane warts (Imahorn et al., 2017). Over the course of the patient’s lifetime, the phenotype of a particular skin area may change, and lesions may progress to NMSC. There is currently no cure for EV. The development of EV lesions cannot be prevented, but frequent examinations of skin lesions that might develop into skin cancers and appropriate treatment of these lesions are recommended, such as surgical removal and cryotherapy. Patients with typical EV are otherwise healthy and are not particularly susceptible to any other unusually severe infectious diseases. Provided that cancers are treated appropriately, life expectancy is similar to that in the general population; multifocal carcinomas might, however, be fatal if untreated. Histological examinations of lesions typically reveal hyperkeratosis and parakeratosis, mild acanthosis, and the presence of koilocytes, keratinocytes with pale-stained cytoplasm in the upper epidermis associated with high levels of intranuclear viral replication. On hematoxylin-eosin staining, the cytoplasm of the affected cells stains pale blue (so-called “blue cells”) and numerous round basophilic keratohyalin granules are visible. This histological finding is pathognomonic for infections with HPV (Orth, 2006). Possible immune infiltration has not been comprehensively characterized in EV lesions. A number of atypical EV cases have been reported, which will be reviewed below.

## Clinical Manifestations of Atypical Ev

Patients with atypical EV have a broader clinical phenotype than those with typical EV. The phenotype of EV, as described above, is undistinguishable between patients with typical and atypical forms. Indeed, the skin lesions develop early in life and are caused by the same beta-HPV subtypes that are found in patients with typical EV (see **Table 1**). The other clinical phenotypes, mostly infectious and auto-immune, differ between patients (Crequer et al., 2012a,b; Sanal et al., 2012; Stray-Pedersen et al., 2014; Stepensky et al., 2015; Li et al., 2016; Platt et al., 2017; Tahiat et al., 2017). The clinical features of patients with atypical EV are reviewed in **Table 1**. Some patients have growth retardation or mild developmental delay. Others present auto-immune features, which may be clinically overt and affect various tissues and organs, or covert, with detectable auto-antibodies but no clinical signs. Bacterial infections, including skin abscesses, pneumonia, and ear infections, are frequent. Infections with cutaneous herpes viruses, molluscum, mucocutaneous candidiasis, and non-beta-HPV infections have also been documented. Moreover, some patients display recurrent respiratory and gastrointestinal infections. Cancers, such as Burkitt lymphoma and EBV-lymphoma, have been reported in some patients. Finally, a few patients have been reported to display clinical manifestations of beta- and alpha-HPV co-infections in different lesions (Azzimonti et al., 2005; Borgogna et al., 2014; Landini et al., 2014). These patients display cutaneous alpha- and beta-HPV- (Borgogna et al., 2014) or genital (Landini et al., 2014) alpha-HPV-driven infections, but no broad susceptibility to infections. These cases suggest that covert immunological abnormalities might favor the development of beta- and alpha-HPV-induced cutaneous and mucosal lesions in other individuals. Based on these clinical observations, the molecular and cellular basis of typical and atypical EV phenotype appear to be different. The typical EV phenotype is, in itself, suggestive of a keratinocyte-intrinsic defect, whereas the atypical EV phenotype, with its myriad of infections and auto-immune features, is more consistent with an adaptive T-cell defect.

**Table 1:** Immunological parameters of patients with typical and atypical EV.

Disease	Genetic etiology^∗^	Clinical phenotypes (affected/total no. of patients)	Expression pattern	T cell counts	T cell function	Other immunological features	Reference
Typical EV	AR TMC6/EVER1	EV	Broad (including keratinocytes and T cells)	Normal	Normal	None	Orth, 2006, 2008; Imahorn et al., 2017
	AR TMC8/EVER2	EV	Broad (including keratinocytes and T cells)	Normal with slightly high proportions for skin-homing subsets	Normal	None	
Atypical EV	AR RHOH deficiency (2 siblings)	Cutaneous viral infections, bronchopulmonary disease, Burkitt lymphoma, **disseminated EV-like flat warts (histologically consistent with EV, HPV-3, -12, and -20 on Southern blotting and PCR)**	Lymphoid lineage	Naïve CD4+ T-cell lymphopenia, high memory CD4+ and CD8+ T-cell counts, low proportions of skin-homing T-cell subsets	Mildly impaired antigen-induced T-cell proliferation, no anti-CD3-induced proliferation	Normal B cell number and function, normal NK cells	Crequer et al., 2012a
	AR MST1 deficiency # (1 patient)	Recurrent respiratory infections, candidiasis, **disseminated** **EV-like flat warts (HPV-5 and -15 positive by Southern blotting and PCR)**, cervical adenopathy, growth retardation	Broad (including keratinocytes and T cells)	Profound CD4+ T-cell lymphopenia (naïve low, memory high), modest naïve and central memory CD8+ lymphopenia, revertant memory CD8+ T-cell counts high	Impaired mitogen (PHA, PMA/Ionomycin)- and antigen (candida, tetanus toxoid, tuberculin)-induced proliferation	Normal B and NK cells, cANCA autoantibodies, high IgA and IgE levels, poor antibody response to several vaccines	Crequer et al., 2012b
	AR CORO1A deficiency # (2 siblings)	Bronchiectasis; fatal EBV-positive lymphoma (1/2); mucocutaneous immunodeficiency syndrome with molluscum contagiosum, oral-cutaneous herpetic (HSV-1) ulcers, **disseminated EV-like HPV infection (HPV-5 and -17 positive by PCR)** and tuberculoid leprosy (1/2)	Broad (including keratinocytes and T cells)	Complete deficiency of naïve CD4+ T cells, high level of double-negative (CD3+CD4-CD8-) gd T cells	Impaired mitogen-induced proliferation, normal antigen-induced proliferation (candida and tetanus toxoid)	T-B +NK+ SCID; absent memory B cells; low NK cell counts, elevated serum IgE, normal serum IgG	Stray-Pedersen et al., 2014
	AR ARTEMIS # p.Leu123Ser (hypomorphic) (1 patient)	Recurrent respiratory and gastrointestinal infections, **persistent disseminated flat warts (histologically consistent with EV, no HPV confirmation)**	broad (including keratinocytes and T cells)	CD4+ T-cell lymphopenia, CD8+ T-cell counts normal	Impaired mitogen- and antigen-induced proliferation	T low B- leaky SCID, IgM levels normal, IgG levels high, IgA absent, normal NK cell counts	Tahiat et al., 2017
	AR DOCK8 deficiency # (4 families)	Recurrent or severe viral infections associated with cancer, atopic dermatitis, recurrent respiratory or gastrointestinal tract infections, disseminated Molluscum contagiosum; disseminated **flat-topped warts (histologically consistent with EV, HPV-5 positive by PCR)**, eczema, hyperpigmentation, and folliculitis	Broad (including keratinocytes and T cells)	CD4+ T-cell lymphopenia	Normal mitogen-induced proliferation	IgM levels low, IgE levels high, variably impaired specific antibody production, normal CD8+ T-cell and B-cell numbers	Sanal et al., 2012; Liu et al., 2017
	AR RASGRP1 # deficiency (1 patient)	Recurrent ear infections, skin abscesses, chronic non-bloody diarrhea, **disseminated warts (histologically consistent with EV, no HPV confirmation)**, severe failure to thrive, splenomegaly, diffuse lymphadenopathy, fatal EBV-positive B cell lymphoma	Broad (including keratinocytes and T cells)	CD4+ T-cell lymphopenia, CD8+ T-cell counts high	Impaired proliferation in response to mitogen (PHA) and antigen (candida and tetanus toxoid)	Normal NK cell number with reduced function, IgG levels low, IgM levels high	Platt et al., 2017
	AR LCK # c.188-2A>G (3 siblings)	Recurrent bacterial pneumonia; **pityriasis-versicolor-like lesions and flat warts on hands, abdomen, legs (histologically consistent with EV, HPV-5, -20, -38 positive by PCR)** and histologically confirmed squamous cell carcinomas, fatal in 1 sibling; genital warts (HPV-6 positive by PCR)	Lymphoid lineage	CD4+ T-cell lymphopenia	Not tested	Not tested	Li et al., 2016
	AR TPP2 deficiency (2 siblings)	Evans syndrome (immune thrombocytopenic purpura and autoimmune hemolytic anemia) (2/2); progressive leukopenia (2/2); mild viral infections (1/2); **flat, hypopigmented warts (HPV-15 positive by PCR) (1/2)**, mild developmental delay (1/2)	Broad (including keratinocytes and T cells)	Normal or slightly low CD4+ T lymphocyte counts	Senescent CD8+ T cells (impaired proliferation, enhanced staurosporine-induced apoptosis)	Premature immunosenescence (T and B cells) and antinuclear antibodies; normal IgA and IgE levels; IgG and IgM levels high	Stepensky et al., 2015

*^∗^Unless the specific mutation is listed, complete deficiency has been verified experimentally. ^#^Other cases with the same genetic etiology but without EV-like symptoms and beta-HPV infections have been described. AR, autosomal recessive+; SCID, severe combined immunodeficiency.*

## Skin Cancer

Patients with EV have a higher than normal risk of developing actinic keratosis and NMSC, particularly cutaneous squamous cell carcinoma (cSCC), cSCC *in situ* (Bowen’s disease) and, to a lesser extent basal cell carcinoma (BCC) (Lewandowsky and Lutz, 1922; Rajagopalan et al., 1972; Orth et al., 1979; Mitsuishi et al., 2008). About two third of EV patients develop NMSC, with an onset during their twenties or thirties (Lewandowsky and Lutz, 1922; Rajagopalan et al., 1972; Orth et al., 1978; Majewski and Jablonska, 1997; de Oliveira et al., 2003). NMSC typically occurs in lesions exposed to the sun, about 20 years after the appearance of the first lesions, suggesting that UV irradiation and HPV are cocarcinogens for the development of this cancer. EV patients should, therefore, pay particular attention to protecting their skin against exposure to the sun. The role of HPVs in carcinogenesis is well established. The first evidence for the oncogenic behavior of HPVs was actually documented in a study of EV (Orth et al., 1980). EV has long served as a human model for studies of viral cutaneous oncogenesis (Jablonska et al., 1972; Casanova and Abel, 2004). This oncogenic behavior of beta-HPVs led to speculations that these viruses might also contribute to the development of cSCC in the general population. However, in cases of cSCC in the general population, less than one viral genome per cell is generally detected; by contrast, viral load is very high in cSCC of EV patients, indicating that different mechanisms of cancer initiation and maintenance are probably at work (Howley and Pfister, 2015). In particular, oncogenic HPV-5 and HPV-8 have been found in EV-associated NMSC, leading to their classification, by the WHO, as possible carcinogens in EV patients (Bouvard et al., 2009). The detection of high loads of HPV-5 E6 and E7 transcripts suggested a role in the cancer induction or maintenance, although the underlying mechanism remains unclear (Orth, 1987). Radiotherapy for cSCC treatment can be effective in the general population, but is counterproductive in EV patients, as the development of more aggressive tumors after treatment has been reported (Rajabi et al., 2014; de Oliveira et al., 2015). Patients should undergo frequent physical examinations, to facilitate the identification of precancerous and cancer lesions as early as possible. However, it has been shown that infection with oncogenic HPVs known to be associated with a high risk of skin cancer is not sufficient, in itself, to cause cancer. Environmental and genetic factors thus contribute to the inter-individual variability of outcome for HPV infection.

## Viral Etiology

More than half a century ago, EV was recognized as a virus-induced disease, based on several experimental observations. Inoculation experiments showed EV to be transmissible (Lutz, 1946; Jablonska and Milewski, 1957; Jablonska et al., 1966). The first viral particles were then detected in lesions by electron microscopy (Ruiter and van Mullem, 1966; Jablonska et al., 1968) and, several years later, the first EV-specific HPV types, all from genus beta-HPV, were isolated from EV lesions (Orth et al., 1978; Orth, 1986). EV patients not only have high HPV loads in their lesions, they also have a high serum antibody reactivity to beta-HPVs (Michael et al., 2010). At least 25 different beta-HPV genotypes have been found in patients with EV, and patients are usually infected with multiple EV-HPV types, HPV-5 being the most prevalent genotype (Orth, 2006). HPV-5 is also the most common genotype in cases of malignant conversion (Orth, 1986; Imahorn et al., 2017). Beta-HPVs are also frequently found in the skin of healthy individuals of the general population, but mostly at low copy numbers, and they do not cause clinical disease. This suggests that beta-HPVs are commensals of the skin (Antonsson et al., 2003a,b). However, it remains a matter of debate whether these beta-HPVs are really non-pathogenic in individuals without EV, as some studies have associated EV-HPV seropositivity and viral DNA load in the eyebrow with the risk of cSCC in the general population (Neale et al., 2013; Iannacone et al., 2014). The mechanisms of persistent, asymptomatic EV-HPV infection remain largely unknown. It has been suggested that beta-HPVs lack an essential growth-promoting function, limiting their pathogenicity. Indeed, unlike the alpha- and gamma-HPVs responsible for cutaneous warts, cervical cancers, and laryngeal papillomatosis, beta-HPVs do not possess the *E5* or *E8* (also reported as E10^1^) open-reading frames typically found in pathogenic non-beta HPVs and shown to be a growth-promoting factor for keratinocytes *in vivo* (Danos et al., 1983; Giri et al., 1985; Hu et al., 2002; Nonnenmacher et al., 2006; Orth, 2006, 2008). Beta-HPVs can overcome this lack of a growth-promoting function and attain full pathogenicity, only in genetically predisposed individuals, implying that these patients have a specific cutaneous immune defect (reviewed by Orth, 2006, 2008).

## Human Genetics of Typical Ev

Typical EV is a Mendelian condition that is transmitted as an autosomal recessive trait and shows complete penetrance in the first decade of life, across different ethnic groups (Orth, 2006). An autosomal recessive mode of inheritance was initially proposed in Cockayne (1933), confirmed in 1974 (Rajagopalan et al., 1972) and subsequently supported by a study of 147 EV cases, in which 11% of patients were the offspring of consanguineous marriages, and 10% came from multiplex families in which 25% of the siblings were affected, with an equal sex ratio (Lutzner, 1978). Shortly after the mapping of two susceptibility loci for EV to chromosomes 17q25 (EV1) and 2p21-p24 (EV2) (Ramoz et al., 1999, 2000), bi-allelic loss-of-function mutations of two adjacent genes termed *TMC6* and *TMC8*, also named *EVER1* and *EVER2*, respectively, located within the EV1 locus, were identified (Ramoz et al., 2002). Eight different mutations of *TMC6/EVER1* and 11 mutations of *TMC8/EVER2* have been described to date, in a total of 32 patients from 19 families and 5 ethnicities reviewed in Burger and Itin (2014) and Imahorn et al. (2017). These mutations are nonsense, frameshift, or splice-site mutations with full clinical penetrance for EV in homozygotes and compound heterozygotes. The effects of these mutations on mRNA and protein levels have not been investigated in most patients. Nonsense-mediated RNA decay and undetectable TMC8/EVER2 protein have been reported in two patients with premature stop codons (Ramoz et al., 2002; Landini et al., 2012). However, splice-site mutations of *TMC8/EVER2* have been identified that have no effect on mRNA levels, despite the detection of aberrantly spliced, shorter transcripts (Miyauchi et al., 2016; Imahorn et al., 2017). Nevertheless, all mutations are assumed to be loss-of-function. By contrast, the missense polymorphisms found in the general population are not predicted to be deleterious and are too common to account for EV. Despite these genetic analyses, more than 40% of the EV families identified to date have no mutations of the genes known to be associated with this disease (Zuo et al., 2006; Arnold et al., 2011; Imahorn et al., 2017) suggesting that high-throughput sequencing studies are required to identify the causal genes in these families.

## Human Genetics of Atypical Ev

Inactivating mutations of *RHOH* (Crequer et al., 2012b)*, STK4* (encoding the MST1 protein) (Crequer et al., 2012a), *CORO1A* (Stray-Pedersen et al., 2014), *DCLRE1C (encoding the Artemis protein)* (Tahiat et al., 2017), *DOCK8* (Sanal et al., 2012; Liu et al., 2017), *RASGRP1* (Platt et al., 2017), *LCK* (Li et al., 2016), and *TPP2* (Stepensky et al., 2015) have been reported in patients with atypical EV. Interestingly, the clinical penetrance of these genetic disorders for EV lesions is low, as only a few patients present with manifestations of EV. This is at odds with the findings for typical EV, which is a fully penetrant Mendelian trait. A complete loss of function has been reported for all mutations, except those for Artemis (hypomorphic) and Lck (no functional investigation). All these patients suffer from a classic T-cell primary immunodeficiency. Some also display impaired mitogen- and/or antigen-induced T-cell proliferation. NK cells counts are normal in all these diseases, except for CORO1A. Hyper-IgM and hyper-IgE are observed in RASGRP1 and in CORO1A and DOCK8, respectively. Autoimmunity has been reported for STK4 and TPP2 deficiencies. T-cell senescence has been reported for RHOH and TPP2 deficiencies. It remains unclear whether these observations are the cause or consequence of severe, disseminated, chronic, life-long beta-HPV and other recurrent acute and chronic infections in these patients. Few studies have addressed the specific responses of leukocytes, but a lack of autologous T-cell response to HPV-infected keratinocytes has been described (Cooper et al., 1990). Most of these genes are broadly expressed (including in T cells and other types of skin-resident cells), with the exception of *RHOH* and *LCK*, which are expressed exclusively in the lymphoid linage (**Table 1**). An additional contribution of cells other than T lymphocytes, including other types of skin-resident cells, to the pathogenesis of atypical EV cannot, therefore, be ruled out. Nevertheless, the T-cell defects underlying atypical EV are likely to be specific, as most inherited and acquired forms of T-cell deficiency do not cause clinically apparent infections with beta-HPVs.

## Leukocyte Immunity

Atypical EV is associated with T-cell deficiency. The only immunological feature common to all known etiologies is CD4^+^ lymphopenia. More detailed immunological studies have been performed only in RhoH-deficient patients. These patients had impaired tissue-homing T-cells marker expression, with a small decreased in the number of memory skin-homing (CLA^+^) CD4^+^ T cells and a large decrease in the number of integrin beta7^+^ CD4^+^ T cells. Broad immunological evaluations of patients with typical EV have proved difficult, due to the rarity of the disease, and the data available are therefore highly patchy. *TMC6/EVER1* and *TMC8/EVER2*, which are mutated in patients with typical EV, are widely expressed, including in lymphocytes and the skin (Lazarczyk et al., 2012). Patients with typical EV have normal humoral immunity and antibody responses (Jablonska et al., 1979), their Langerhans cells have normal alloantigen presentation capacity (Majewski and Jablonska, 1992), and they have largely normal NK cell activity (Majewski et al., 1986). They have been reported to display impairments of cellular immunity, including low T-cell counts, CD4^+^/CD8^+^ T-cell ratios, a lack of responsiveness to T-cell mitogens, anergy to skin antigens and sensitization to dinitrochlorobenzene (Glinski et al., 1976, 1981; Prawer et al., 1977; Majewski et al., 1986; Majewski and Jablonska, 1992; Majewski et al., 1997; de Oliveira et al., 2003), but these defects were inconsistent and further complicated the assessment of immunological dysfunction in EV patients. All these observations suggest that a role for TMC6/EVER1 and TMC8/EVER2 in skin-intrinsic immunity.

## Keratinocyte Immunity

Keratinocytes contribute to protective immunity in the skin in at least three ways. First, they form a physical barrier. Second, they secrete and respond to cytokines, chemokines, and growth factors, participating in leukocyte-mediated operations (Kupper and Fuhlbrigge, 2004). Third, keratinocyte-intrinsic mechanisms are thought to contribute to the control of keratinocyte-tropic viruses. TMC6/EVER1 and TMC8/EVER2 thus provide us a unique opportunity to study the role of keratinocytes in host defense against HPV infection. TMC6/EVER1 and TMC8/EVER2 belong to a large family of highly conserved transmembrane channel-like (TMC) proteins that are expressed in the endoplasmic reticulum, where they act as or modulate the activity of transmembrane channels (Keresztes et al., 2003; Kurima et al., 2003). It has been suggested that these two proteins interact with the zinc transporter ZnT-1, influencing intracellular Zn^2+^ concentration, and, thus, the activity of the transcription factors, such as AP-1, a key activator in the HPV life cycle and cellular proliferation (Lazarczyk et al., 2008). It has also been suggested that TMC8/EVER2 influences the cellular response to TNF-α by promoting apoptosis rather than NF-κB activation and pro-survival signaling pathways (Gaud et al., 2013; Vuillier et al., 2014). TMC6/EVER1 and TMC8/EVER2 are thus thought to serve as restriction factors for beta-HPVs in keratinocytes, through the limitation of viral replication and gene expression. Studies of TMC6/EVER1 and TMC8/EVER2 have proved technically difficult, partly due to problems raising robust antibodies against these highly hydrophobic proteins, which have 10 and 8 transmembrane domains, respectively. Known molecular functions/mechanism of TMC6/EVER1 and TMC8/EVER2 are reviewed in Orth (2008) and Lazarczyk et al. (2009). The cellular and molecular bases of beta-HPV-driven lesions in TMC6/EVER1 and TMC8/EVER2-deficient patients with EV remain unknown. The genetic defects prevent patients from controlling beta-HPV in keratinocytes (Akgul et al., 2006). The patients are susceptible to skin infections with these particular types of HPV (Boxman et al., 1999). In addition, some patients with severe combined immunodeficiency (SCID) caused by mutations of *IL2RG* or *JAK3* may develop EV. These patients develop EV only after successful bone marrow transplantation, indicating that disease development in these patients is related to a keratinocyte defect (Laffort et al., 2004).

## A Classification of Ev Into Two Major Types

In the past, EV patients rarely underwent immunological follow-up, and typical and atypical cases were treated as the same diagnostic entity. This has led to conflicting reports about the impairment of immunity in only some patients. Patients with typical EV probably have a keratinocyte-intrinsic defect allowing beta-HPVs to persist in the skin, leading to the development of clinically apparent lesions. Consistent with this notion, three TMC8/EVER2-deficient EV patients have been reported to display a largely normal partitioning of circulating T cells, possibly even with slightly larger than normal skin-homing T-cell populations (Crequer et al., 2013). These normal numbers of circulating T cells do not exclude the possibility of an impaired recognition of and response to HPV-infected keratinocytes by these cells (Cooper et al., 1990). Once it became clear that these patients had a selective susceptibility to beta-HPV infections and TMC6/EVER1 and TMC8/EVER2 deficiencies had been described as a genetic etiology, EV was identified as a primary immunodeficiency (Notarangelo et al., 2004; Orth, 2008; Casanova, 2015). It has been suggested that the TMC/EVER proteins function as antiviral restriction factors in keratinocytes, and that they control constitutive intrinsic immunity (Orth, 2008). Consistent with this hypothesis, patients with acquired or treatment-induced immunosuppression do not normally develop EV, despite the commensal nature of beta-HPVs (Antonsson et al., 2003a,b). This contrasts with common warts due to alpha-HPVs, which are more common in immunosuppressed individuals (Wieland et al., 2014). Rare cases of EV-like syndromes developing in previously unaffected adults, and known as “acquired EV,” have been described in patients with suppressed cell-mediated immunity. The clinical presentations of these patients and proposed treatments have been reviewed in detail elsewhere (Zampetti et al., 2013; Ovits et al., 2017). The rarity of atypical EV in patients with broad and severe genetic T-cell deficiencies and in immunocompromised individuals suggests that immunosuppression *per se* is not sufficient for the development of this disease. This is the case for most infectious diseases striking patients with such T-cell disorders, and it suggests that other host and environmental factors are involved in pathogenesis.

## Concluding Remarks

In summary, both keratinocytes and T cells probably contribute to immunity to beta-HPV. Genetic defects affecting one or, potentially, both cell types lead to the EV-defining clinical manifestations of beta-HPV infection. All individuals with severe, recurrent, and persistent flat warts and with a diagnosis of typical or atypical EV should be tested for genetic etiologies of typical and atypical EV. The presence and extent of a systemic T-cell defect determines the spectrum of other clinical manifestations of an infectious, autoimmune, or allergic nature. Future studies should provide more conclusive demonstrations of the keratinocyte-intrinsic nature of EVER-dependent immunity to beta-HPVs. The identification of new genes associated with typical and atypical EV will pave the way for analyses of the molecular and cellular bases of EV, including studies of the respective contributions of keratinocytes and T cells to anti-beta-HPV immunity. The highly restricted susceptibility to beta-HPV observed in patients with EV has important clinical and biological implications in the fields of HPV, mucocutaneous immunology, and cell-intrinsic immunity. Elucidation of the genetic and immunological bases of EV will facilitate studies of the virulence mechanisms of other HPVs, including the role of E5 and E8 proteins produced by alpha- and gamma-HPVs, respectively. These studies will also facilitate the development of novel approaches for the diagnosis and management of patients with various conditions due to HPVs.

## Author Contributions

SdJ, EI, PI, GO, EJ, J-LC, and BB wrote the manuscript. SdJ, EI, PI, JU, GO, EJ, J-LC, and BB reviewed and edited the manuscript.

## Conflict of Interest Statement

The authors declare that the research was conducted in the absence of any commercial or financial relationships that could be construed as a potential conflict of interest. The reviewer GS and handling Editor declared their shared affiliation.

## Footnotes

1 http://PAVE.niaid.nih.gov
